# Risk Factors for Vitamin D Deficiency among Veterans with and without HIV Infection

**DOI:** 10.1371/journal.pone.0124168

**Published:** 2015-04-21

**Authors:** Alicia I. Hidron, Brittany Hill, Jodie L. Guest, David Rimland

**Affiliations:** 1 Atlanta Veterans Affairs Medical Center, Decatur, Georgia, United States of America; 2 Emory University School of Medicine, Atlanta, Georgia, United States of America; 3 Universidad Pontificia Bolivariana, Medellín, Colombia; 4 Hospital Pablo Tobón Uribe, Medellín, Colombia; 5 Winship Cancer Institute at Emory University, Atlanta, Georgia, United States of America; 6 Rollins School of Public Health at Emory University, Atlanta, Georgia, United States of America; University of Tennessee, UNITED STATES

## Abstract

**Objectives:**

We aimed to describe and compare the prevalence of vitamin D deficiency between HIV-negative and HIV-infected veterans in the southern United States, and to determine risk factors for vitamin D deficiency for HIV infected patients.

**Methods:**

Cross-sectional, retrospective study including all patients followed at the Atlanta VA Medical Center with the first 25-hydroxyvitamin D [25(OH)D] level determined between January 2007 and August 2010. Multivariate logistic regression analysis was used to determine risk factors associated with vitamin D deficiency (< 20 ng/ml).

**Results:**

There was higher prevalence of 25(OH)D deficiency among HIV-positive compared to HIV-negative patients (53.2 vs. 38.5%, p <0.001). Independent risk factors for vitamin D deficiency in HIV + patients included black race (OR 3.24, 95% CI 2.28–4.60), winter season (OR 1.39, 95% CI 1.05–1.84) and higher GFR (OR 1.01, CI 1.00–1.01); increasing age (OR 0.98, 95% CI 0.95–0.98), and tenofovir use (OR 0.72, 95% CI 0.54–0.96) were associated with less vitamin D deficiency.

**Conclusions:**

Vitamin D deficiency is a prevalent problem that varies inversely with age and affects HIV-infected patients more than other veterans in care. In addition to age, tenofovir and kidney disease seem to confer a protective effect from vitamin D deficiency in HIV-positive patients.

## Introduction

It has long been known that vitamin D plays an important role in skeletal health. [1] Serum levels of vitamin D change with the seasons, exposure to sunlight and dietary intake and are inversely related to skin pigmentation and body mass index (BMI). [1–3] Vitamin D associations to extraskeletal outcomes such as cancer, cardiovascular disease, diabetes, autoimmune diseases and infections have also been reported. [4,5] The evidence to support the latter associations does not come from randomized controlled trials so that the Institute of Medicine considers levels lower than 20 ng/ml as deficient based entirely on skeletal outcomes. [1] In contrast, considering evidence on skeletal as well as extraskeletal disease, the US Endocrine Society recommended that vitamin D deficiency be defined as a 25(OH)D level of 20 ng/mL or less and vitamin D insufficiency as 21 to 29 ng/mL for children and adults. [6]

Vitamin D is a key player in the innate immune system and in immune modulation; thus, it has been evaluated for prevention and treatment of different infections. [7] Its role in HIV disease is unclear, but deficiency of this vitamin has been reported with increasing frequency in HIV-infected patients. [8–11] Despite the high prevalence of vitamin D deficiency in this group of patients, it has been reported to be lower than that of the general population. [12,13] In HIV-infected patients vitamin D levels may also depend on HIV treatment; efavirenz use is associated with lower levels [8,12] while tenofovir use has been associated with higher levels of this vitamin. [12]

Our aims were to describe and compare the prevalence of vitamin D deficiency between HIV-negative and HIV-infected patients in a single healthcare system, and to determine risk factors for vitamin D deficiency in this population.

## Methods

This cross-sectional, retrospective study included all patients followed at the Atlanta VA Medical Center (VAMC) with the first 25-hydroxyvitamin D [25(OH)D] level determined between January 2007 and August 2010. 25(OH)D determinations were obtained as part of routine clinical care during this time period. This study had Institutional Review Board (IRB) approval from Emory University and the AtlantaVeteran´s Affairs Medical Center. All patients followed at the HIV AtlantaVeteran´s Affairs Medical Center Cohort Study (HAVACS) with a vitamin D level during this same time frame were also included. HAVACS database has had an IRB waiver from Emory University and the Atlanta VAMC since 1982. Patient records and information were anonymized and de-identified prior to analysis.

Specific demographic, clinical and laboratory data were obtained from electronic medical records. The liquid chromatography/tandem mass spectrometry method was used for measuring 25(OH)D (Quest Laboratories). 25(OH)D deficiency and insufficiency was defined as a level of <20 and ≥20–<30 ng/ml, respectively, based on previous assessments and published optimal Vitamin D levels for preventing adverse outcomes. [1,2,14–16] Standard, routine laboratory assays were used to measure serum chemistry tests. The glomerular filtration rate (GFR) was estimated by the Cockroft-Gault equation.

For the statistical analysis, Pearson’s exact chi square and Wilcoxon two-sided tests were used for categorical and continuous variables, respectively. Variables examined included: age, gender, race, body mass index (BMI), month of the year the 25(OH)D levels were obtained (season) and HIV status. For the HIV-infected cohort, additional variables included specific risk factor for HIV, years since HIV diagnosis, nadir CD4 count, history of opportunistic infections, comorbidities, lab values (creatinine, parathyroid hormone (PTH), calcium, phosphorus levels, current CD4 T cell count, current HIV viral load), and antiretroviral (ARV) regimen. Variables that were significant on univariate analysis were included in a multivariate logistic regression model to determine independent risk factors associated with vitamin D deficiency. A p-value of <0.05 was used to determine variables that remained significantly associated with Vitamin D deficiency. SAS (version 9.2; SAS Institute, Cary, NC) software was used to perform the analyses.

## Results

### Total VA Population

**Fig 1:**
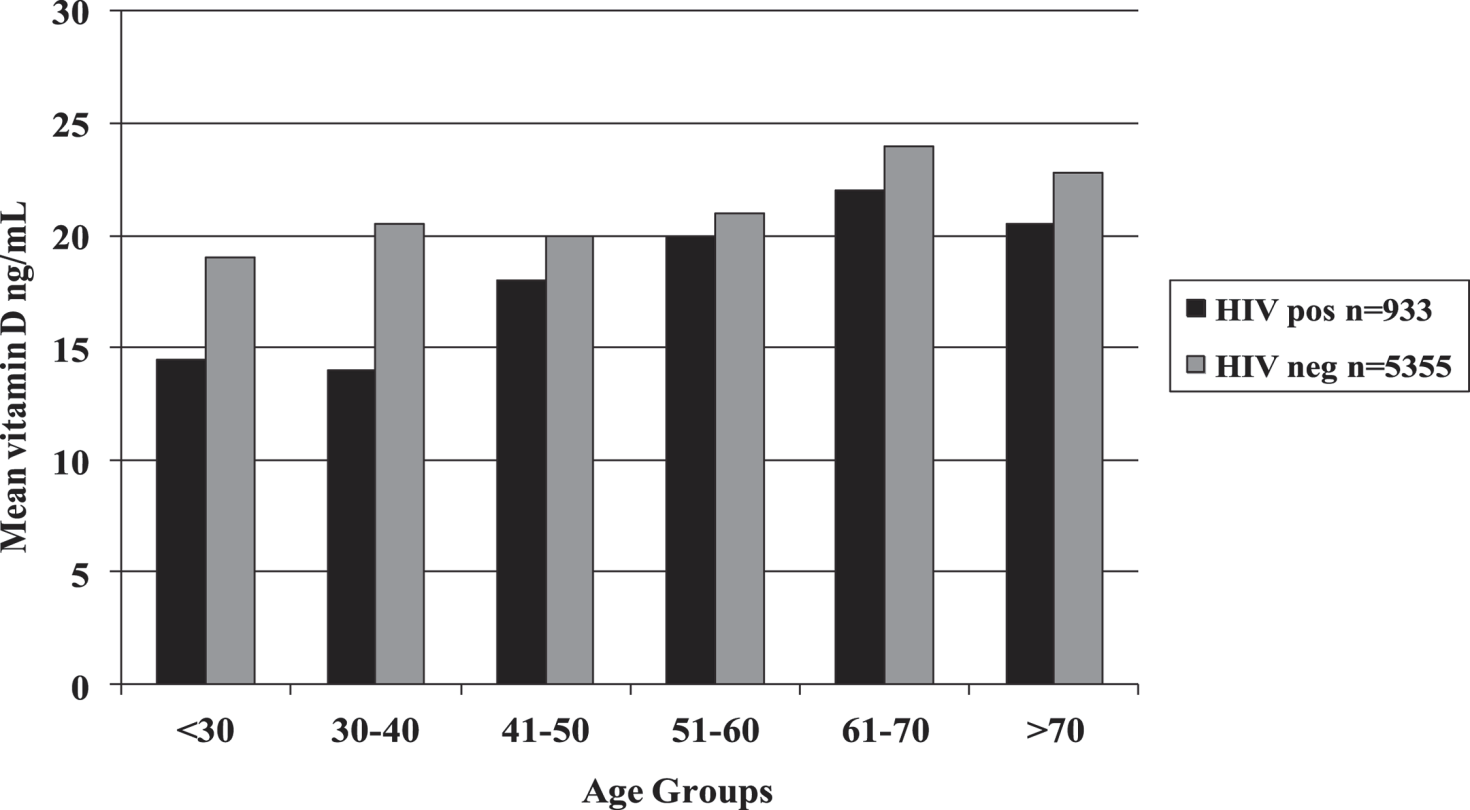
Mean Vitamin D Level by Age Group for HIV positive and HIV negative Patients.

A total of 6288 veterans were included in the study. The overall prevalence of 25(OH)D deficiency was 40.7% (2558/6288). Median age was 61 years (range 22–97), 50.1% were black, and 88.5% male. The median BMI was 26. Close to 15% of the patients were HIV-infected and 55.7% of the 25(OH)D levels were drawn during the winter months. Significant associations with 25(OH)D deficiency in univariate analysis included: younger median age (59 vs. 63, p<0.0001), black race (68.5 vs. 37.3%, p<0.0001), male gender (86.9 vs. 89.6%, p 0.0011), higher median BMI (27 vs. 26, p = 0.0005), HIV-positive status (19.4 vs. 11.7%, p<0.001), and levels drawn during the winter season (61.7 vs. 51.5%, p<.0001) (Table 1). There was higher prevalence of 25(OH)D deficiency among HIV-positive compared to HIV-negative patients (53.2% (95% CI: 51.6, 54.8) vs. 38.5% (95% CI: 37.2, 39.8), p <0.001); this was true for all age groups (Fig 1). Median 25(OH)D levels were 24 ng/ml for HIV-negative patients and 19 ng/ml for HIV positive patients (p<0.001). HIV positive patients were also more likely African American, younger, male and less obese (Table 2). In multivariate analysis, independent risk factors for 25(OH)D deficiency included black race (odds ratio (OR) 3.34, 95% confidence interval (CI) 2.98–3.74), higher BMI (OR 1.02, 95% CI 1.01–1.03), and winter season (OR 1.70, 95% CI 1.52–1.90). There was a significant interaction between age and HIV status so that for each additional decade of life, people who were HIV-negative had an 8% reduction in the odds of 25(OH) D deficiency (OR 0.92, 95% CI 0.88–0.96 for every decade of life) compared to a 29% reduction for HIV-positive patients (OR 0.71, 95% CI 0.62–0.82 for every decade of life) (Table 3).

**Table 1:** Risk factors associated with Vitamin D deficiency, all patients; univariate analysis.

**Risk Factor**	**Vit D < 20 (n = 2558)**	**Vit≥20D (n = 3730)**	**P value**
Median age (range)	59 (22–95)	63 (22–97)	<.0001
Black race n (%)	1752 (68.5)	1394 (37.3)	<.0001
Male gender n (%)	2224 (86.9)	3343 (89.6)	0.0011
Median BMI* (range)	27 (12–62)	26 (14–54)	0.0005
HIV infection n (%)	496 (19.4)	437 (11.7)	<.0001
Winter/spring season n (%)	1579 (61.7)	1921 (51.5)	<.0001

**Table 2:** Characteristics of HIV-negative versus HIV positive patients.

**Characteristic**	**HIV negative (n = 5355)**	**HIV positive (n = 933)**	**P value**
Age median (range)	63 (22–97)	50 (24–86)	<.0001
BMI median (range)	26 (12–62)	26 (15–53)	0.0002
Black race n (%)	2425 (45.3)	721 (77.4)	<.0001
Vitamin D <20 ng/ml n (%)	2062 (38.5)	495 (53.2)	<.0001
Male gender n (%)	4655 (86.9)	910 (97.5)	<.0001
Winter season n (%)	3112 (58.1)	388 (41.6)	<.0001

**Table 3:** Risk factors associated with Vitamin D deficiency, all patients; multivariate analysis.

**Risk factor**	**OR (95% CI)**
Black race	3.34 (2.98–3.74)
Winter/spring season	1.70 (1.52–1.90)
BMI	1.02 (1.01–1.03)
10-year increase in age	
among HIV negative	0.92 (0.88–0.96)
among HIV positive	0.71 (0.62–0.82)

### HIV-positive population only

A total of 933 HIV infected patients were studied. The overall prevalence of 25(OH)D deficiency was 53.2% (496/933). Median age was 50 years (24–86), 77.3% were black, and 53.5% men who have sex with men (MSM). The median CD4 count and viral load at the time of the 25(OH)D level were 466 (2–2103) and <48 copies, respectively. 56.7% of patients had an undetectable viral load; only 12.9% of patients had CD4 counts <200 and 82% patients were on ARVs. The most frequent comorbid conditions included: obesity (BMI ≥ 25) (56.2%), hypertension (50.4%), smoking (44.1%), hepatitis C (17.9%), diabetes (14.4%), chronic kidney disease stage III or worse (GFR ≤ 60) (10.5%), cardiovascular disease (9.4%) and hepatitis B (8.4%). Significant associations with 25(OH)D deficiency in univariate analysis included: younger median age (48 vs. 52, p<0.0001), black race (87.7 vs. 66.2%, p<0.0001), higher median GFR (93 vs. 86, p<0.0001), hepatitis B (10.1 vs. 6.4%, p = 0.04), median number of years since HIV diagnosis (10.9 vs. 12.5, p = 0.02), winter season (45.4 vs. 37.3%, p = 0.01), undetectable viral load (53.1 vs. 60.6%, p = 0.02), tenofovir use (57.7 vs. 65.9%, p = 0.01), protease inhibitor use (32.9 vs. 43.0%, p = 0.002) and no ARV use (21.2 vs. 13.0%, p = 0.001) (Table 4). In multivariate analysis, independent risk factors for 25(OH)D deficiency included black race (OR 3.30, 95% CI 2.30–4.74), higher GFR (OR 1.01, 95% CI 1.00–1.01) and winter season (OR 1.37, 95% CI 1.03–1.83). Older age (OR 0.97, 95% CI 0.95–0.99) and tenofovir use (OR 0.58, 95% CI 0.37–0.89) were associated with less 25(OH)D deficiency (Table 5).

**Table 4:** Risk factors associated with Vitamin D deficiency, HIV-positive patients; univariate analysis.

**Risk Factor**	**Vitamin D <20 (n = 496) n/N (%)**	**Vitamin≥20 D (n = 437) n/N (%)**	**P value**
Age (median, range)	48 (25–77)	52 (24–86)	<0.0001
Race			
Black	434/723 (60.0)	289/723 (40.0)	<0.0001
Other	61/208 (29.3)	147/208 (70.7)	
Risk factor			
MSM	265/499 (53.1)	234/499 (46.9)	0.50
IVDU	44/92 (47.8)	48/92 (52.2)	
Other	187/342 (54.7)	155/342 (45.3)	
Comorbidities			
GFR (median, range)	93 (5.6–185.7)	86 (11.7–176.7)	<0.0001
BMI (median, range)	25.8 (14.8–47.7)	25.3 (15.6–52.5)	0.15
Current smoker	222/411 (54.0)	186/411 (46.0)	0.64
Hypertension	257/470 (54.7)	213/470 (45.3)	0.36
Cardiovascular disease	48/88 (54.6)	40/88 (45.4)	0.82
Diabetes	69/134 (51.5)	65/134 (48.5)	0.71
Hepatitis C	78/167 (46.7)	89/167 (53.3)	0.07
Hepatitis B	50/78 (64.1)	28/78 (35.9)	0.04
HIV Factors			
Years since HIV diagnosis	10.9 (-0.7–29.0)	12.5 (0.0–26.2)	0.002
Nadir CD4	173.5 (0–978)	159.5 (0–1034)	0.1
History of OI	138/261 (52.9)	123/261 (47.1)	0.94
CD4 count	466.5 (2–1460)	466 (5–2103)	0.4
Viral load log ≥1.68	232/404 (57.4)	172/404 (42.3)	0.02
CD4 delta since diagnosis	235 (0.0–1210)	278 (0.0–2075)	0.02
Current ARV regimen			
None	107/167 (64.1)	60/167 (35.9)	0.0007
TDF with EFV	162/295 (54.9)	133/295 (45.1)	
TDF without EFV	124/279 (44.4)	155/279 (55.6)	
Any regimen without TDF	103/192 (53.7)	89/192 (46.4)	
Lab values			
Median PTH	58 (13.4–592.5)	45.7 (4.5–1180)	<0.0001
Median calcium level	9.2 (5.4–10.6)	9.3 (8.0–12.3)	<0.0001
Median phosphorus level	3.2 (1.2–7.5)	3.3 (1.3–5.3)	0.18
Season			
Summer/Fall	271/545 (49.7)	274/545 (50.3)	0.01
Winter/Spring	225/388 (58.0)	163/388 (42.0)	

**Table 5:** Risk factors associated with Vitamin D deficiency, HIV-positive patients; multivariate analysis.

**Risk Factor**	**OR (95% CI)**
Black race	3.30 (2.30–4.74)
Higher GFR*	1.01 (1.00–1.01)
Winter/spring season	1.37 (1.03–1.83)
Advancing age*	0.97 (0.95–0.99)
Treatment **	
No ARV	1
Tenofovir use with efavirenz	0.86 (0.55–1.33)
Tenofovir use without efavirenz	0.58 (0.37–0.89)
Any other ARV regimen without TDF	0.91 (0.57–1.47)

*Odds of Vitamin D deficiency is 0.73 for every 10-year increase in age and 1.21 for every 25-point increase in GFR

**at the time of Vitamin D level

## Discussion

We found a high prevalence of vitamin D deficiency among veterans in care in Atlanta. Risk factors for 25(OH)D deficiency included black race, higher BMI and winter season. These associations have been reported previously and derive from decreased synthesis of vitamin D in skin due to darker skin pigmentation and lack of sunlight exposure and/or exercise during the winter months and among obese people. [2,4,17]

Aside from these known associations, age seemed to confer a protective effect, which was more pronounced among HIV-positive patients. For each additional decade of life, people who were HIV-negative had an 8% reduction in the odds of vitamin D deficiency compared to a 29% reduction for HIV-positive patients. The changes in vitamin D status with increasing age are controversial. Original data from NHANES III documented that advancing age was associated with lower levels of 25(OH)D3, [18] but more recent data from NHANES 2001–2004 showed no difference in 25(OH)D by age from 12 to over 60. [14] Data from the Women’s Interagency HIV Study (WIHS) found an association between increasing age and higher vitamin D levels in women. [19] Although African American patients were overall younger among both HIV-positive and negative patients, the multivariate model controlled for race, so that racial differences do not entirely explain this unexpected finding.

In a predominantly male, African-American, outpatient cohort of veterans we found a higher prevalence of Vitamin D deficiency among HIV-positive compared to HIV-negative patients (53.2 vs. 38.5%). Ormesher et al and Dao et al found a higher prevalence of deficiency among HIV-negative patients, but they used an age-matched control group from NHANES data. [12,20] This type of matching may not account for other differences in race, geographic location or other socioeconomic factors. Few studies have compared HIV-positive to HIV-negative patients with similar demographic factors. A recent report from the WIHS demonstrated more vitamin D deficiency in HIV-positive women compared to HIV-negative women.[18] As supported by other studies,[9] these differences in vitamin D deficiency are probably not related to the patients' underlying immune status as the majority of our patients had good CD4 counts, undetectable viral loads and were receiving HAART. Furthermore, immune status (CD4 count) and virologic status (HIVviral load) were not found to be markers for vitamin D deficiency in HIV-positive patients. It is plausible that this difference could be driven by overrepresentation of young, comparatively slimmer African American males in our HIV cohort. However, HIV infection remained as an independent association after controlling for age, BMI, race, season and gender in a multivariate model.

For HIV-positive patients, we confirmed the association of vitamin D deficiency and black race,[11,12,21–24] higher GFR, [12] and season. [12,17,21–23] Consistent with some published data, we identified less vitamin D deficiency in patients on tenofovir (TDF) and patients with mild renal insufficiency.[12] The proportion of patients on ART receiving TDF was not significantly different between patients with and without vitamin D deficiency (36.8% vs. 37.6% respectively). However, 21.1% of the patients on TDF that had vitamin D deficiency were receiving efavirenz (EFV) concomitantly in contrast to only 17.4% of those not receiving EFV with TDF. EFV has been shown to be associated with vitamin D deficiency. [8,12] Since a protective effect for tenofovir was only observed for patients also not on Efavirenz, it is plausible that what we are observing could be explained by the deleterious effect of Efavirenz on lowering Vitamin D levels more than that of a protective effect of TDF. Alternatively, TDF has been associated with secondary hyperparathyroidism and osteomalacia. [25,26] The elevation in PTH caused by phosphate renal loss (which is seen with TDF) [27] could also be mediating the observed relative increase in Vitamin D levels. In this study we did not examine the dynamic interplay between Vitamin D levels, PTH, renal function and TDF. These interactions—which might explain our observations as well as others’- are complex, poorly understood, and will require further research.

Our study confirms several of the known associations with Vitamin D deficiency. The strengths of this study include a large patient population and the ability to identify and compare risk factors among HIV positive and negative patients in a similar population based on electronic data. Some limitations need to be noted. First, we lacked patient specific data on the HIV negative patients including vitamin D dietary and supplementation history. The use of the first measured vitamin D level should minimize any bias. Second, our study may not be generalizable to other patient populations since it represents a single site study in the southern U.S. and involved mostly males. Finally, due to the cross-sectional nature of the data, we want to point out that our findings demonstrate only associations. However, prospective studies have proven associations similar to the ones reported in this paper, as well as associations with outcomes such as an increased risk of virologic failure, HIV progression and even death. [17,28]

In summary, we identified vitamin D deficiency as a prevalent problem affecting HIV-infected patients more than other veterans in care. This deficiency is ameliorated by aging in both HIV-positive and negative veterans. In addition to age, tenofovir and kidney disease seem to confer a protective effect from vitamin D deficiency in HIV-positive patients. The clinical impact of these observations will need to be prospectively studied.

## References

[1] Institute of Medicine 2011 Dietary reference intakes for calcium and vitamin D Washington, D.C.: The National Academy Press.

[2] Rosen CJ. Clinical practice. Vitamin D insufficiency. The New England journal of medicine. 2011;364(3):248–54. 10.1056/NEJMcp1009570 21247315

[3] Zadshir A, Tareen N, Pan D, Norris K, Martins D. The prevalence of hypovitaminosis D among US adults: data from the NHANES III. Ethnicity & disease. 2005;15(4 Suppl 5):S5-97-101. 16315387

[4] Holick MF. Vitamin D deficiency. The New England journal of medicine. 2007;357(3):266–81. 17634462 10.1056/NEJMra070553

[5] Hossein-nezhad A, Holick MF. Vitamin D for health: a global perspective. Mayo Clinic proceedings. 2013;88(7):720–55. Epub 2013/06/25. 10.1016/j.mayocp.2013.05.011 23790560 PMC3761874

[6] Holick MF, Binkley NC, Bischoff-Ferrari HA, Gordon CM, Hanley DA, Heaney RP, et al Evaluation, treatment, and prevention of vitamin D deficiency: an Endocrine Society clinical practice guideline. The Journal of clinical endocrinology and metabolism. 2011;96(7):1911–30. Epub 2011/06/08. 10.1210/jc.2011-0385 21646368

[7] Schwalfenberg GK. A step in the right direction. CMAJ: Canadian Medical Association journal = journal de l'Association medicale canadienne. 2010;182(16):1763. 10.1503/cmaj.110-2118 PMC2972334 21059788

[8] Welz T, Childs K, Ibrahim F, Poulton M, Taylor CB, Moniz CF, et al Efavirenz is associated with severe vitamin D deficiency and increased alkaline phosphatase. AIDS (London, England). 2010;24(12):1923–8. 10.1097/QAD.0b013e32833c3281 20588161

[9] Rosenvinge MM, Gedela K, Copas AJ, Wilkinson A, Sheehy CA, Bano G, et al Tenofovir-linked hyperparathyroidism is independently associated with the presence of vitamin D deficiency. Journal of acquired immune deficiency syndromes (1999). 2010;54(5):496–9. 20672448 10.1097/qai.0b013e3181caebaa

[10] Garcia Aparicio AM, Munoz Fernandez S, Gonzalez J, Arribas JR, Pena JM, Vazquez JJ, et al Abnormalities in the bone mineral metabolism in HIV-infected patients. Clinical rheumatology. 2006;25(4):537–9. 16208429 10.1007/s10067-005-0028-x

[11] Crutchley RD, Gathe JC, Mayberry C, Trieu A, Abughosh S, Garey K. Risk Factors for Vitamin D Deficiency in HIV-Infected Patients in the South Central United States. AIDS Res Hum Retroviruses. 2011. Epub 2011/09/01. 10.1089/aid.2011.0025 21878055

[12] Dao CN, Patel P, Overton ET, Rhame F, Pals SL, Johnson C, et al Low vitamin D among HIV-infected adults: prevalence of and risk factors for low vitamin D Levels in a cohort of HIV-infected adults and comparison to prevalence among adults in the US general population. Clinical infectious diseases: an official publication of the Infectious Diseases Society of America. 2011;52(3):396–405. 21217186 10.1093/cid/ciq158

[13] Van Den Bout-Van Den Beukel CJ, Fievez L, Michels M, Sweep FC, Hermus AR, Bosch ME, et al Vitamin D deficiency among HIV type 1-infected individuals in the Netherlands: effects of antiretroviral therapy. AIDS research and human retroviruses. 2008;24(11):1375–82. 10.1089/aid.2008.0058 18928396

[14] Ginde AA, Liu MC, Camargo CAJCINNRRA, Pmid. Demographic differences and trends of vitamin D insufficiency in the US population, 1988–2004. Archives of internal medicine. 2009;169(6):626–32. 10.1001/archinternmed.2008.604 19307527 PMC3447083

[15] Dawson-Hughes B, Heaney RP, Holick MF, Lips P, Meunier PJ, Vieth R. Estimates of optimal vitamin D status. Osteoporosis international: a journal established as result of cooperation between the European Foundation for Osteoporosis and the National Osteoporosis Foundation of the USA. 2005;16(7):713–6. 10.1007/s00198-005-1867-7 15776217

[16] Bischoff-Ferrari HA, Giovannucci E, Willett WC, Dietrich T, Dawson-Hughes B. Estimation of optimal serum concentrations of 25-hydroxyvitamin D for multiple health outcomes. The American journal of clinical nutrition. 2006;84(1):18–28. 16825677 10.1093/ajcn/84.1.18

[17] Havers F, Smeaton L, Gupte N, Detrick B, Bollinger RC, Hakim J, et al 25-Hydroxyvitamin D insufficiency and deficiency is associated with HIV disease progression and virological failure post-antiretroviral therapy initiation in diverse multinational settings. J Infect Dis. 2014;210(2):244–53. Epub 2014/05/07. 10.1093/infdis/jiu259 24799602 PMC4141201

[18] Zadshir A, Tareen N, Pan D, Norris K, Martins D. The prevalence of hypovitaminosis D among US adults: data from the NHANES III. Ethn Dis. 2005;15(4 Suppl 5):S5-97-101. Epub 2005/12/01. 16315387

[19] Adeyemi OM, Agniel D, French AL, Tien PC, Weber K, Glesby MJ, et al Vitamin D deficiency in HIV-infected and HIV-uninfected women in the United States. J Acquir Immune Defic Syndr. 2011;57(3):197–204. Epub 2011/04/08. 10.1097/QAI.0b013e31821ae418 21471818 PMC3431159

[20] Ormesher B, Dhaliwal S, Nylen E, Gibert C, Go C, Amdur R, et al Vitamin D deficiency is less common among HIV-infected African-American men than in a matched cohort. AIDS. 2011;25(9):1237–9. Epub 2011/04/21. 10.1097/QAD.0b013e3283474ef9 21505299

[21] Vescini F, Cozzi-Lepri A, Borderi M, Re MC, Maggiolo F, De Luca A, et al Prevalence of Hypovitaminosis D and Factors Associated With Vitamin D Deficiency and Morbidity Among HIV-Infected Patients Enrolled in a Large Italian Cohort. Journal of acquired immune deficiency syndromes (1999). 2011;58(2):163–72. 10.1097/QAI.0b013e31822e57e9 21826011

[22] Fox J, Peters B, Prakash M, Arribas J, Hill A, Moecklinghoff C. Improvement in vitamin D deficiency following antiretroviral regime change: Results from the MONET trial. AIDS research and human retroviruses. 2011;27(1):29–34. 10.1089/aid.2010.0081 20854196

[23] Viard JP, Souberbielle JC, Kirk O, Reekie J, Knysz B, Losso M, et al Vitamin D and clinical disease progression in HIV infection: results from the EuroSIDA study. AIDS. 2011;25(10):1305–15. Epub 2011/04/28. 10.1097/QAD.0b013e328347f6f7 21522006

[24] Kim JH, Gandhi V, Psevdos G, Espinoza F, Park J, Sharp V. Evaluation of Vitamin D Levels among HIV-Infected Patients in New York City. AIDS research and human retroviruses. 2011. 10.1089/AID.2011.0040 21644847

[25] Childs KE, Fishman SL, Constable C, Gutierrez JA, Wyatt CM, Dieterich DT, et al Short communication: Inadequate vitamin D exacerbates parathyroid hormone elevations in tenofovir users. AIDS research and human retroviruses. 2010;26(8):855–9. 10.1089/aid.2009.0308 20672993 PMC2957627

[26] Gallant JE, Staszewski S, Pozniak AL, DeJesus E, Suleiman JM, Miller MD, et al Efficacy and safety of tenofovir DF vs stavudine in combination therapy in antiretroviral-naive patients: a 3-year randomized trial. JAMA: the journal of the American Medical Association. 2004;292(2):191–201. 15249568 10.1001/jama.292.2.191

[27] Woodward CL, Hall AM, Williams IG, Madge S, Copas A, Nair D, et al Tenofovir-associated renal and bone toxicity. HIV medicine. 2009;10(8):482–7. 10.1111/j.1468-1293.2009.00716.x 19459988

[28] Shepherd L, Souberbielle JC, Bastard JP, Fellahi S, Capeau J, Reekie J, et al Prognostic value of vitamin D level for all-cause mortality, and association with inflammatory markers, in HIV-infected persons. J Infect Dis. 2014;210(2):234–43. Epub 2014/02/05. 10.1093/infdis/jiu074 24493824

